# Postdoctoral scientists are mentors, and it is time to recognize their work

**DOI:** 10.1371/journal.pbio.3002349

**Published:** 2023-11-02

**Authors:** Gracielle Higino, Ceres Barros, Ellen Bledsoe, Dominique G. Roche, Sandra Ann Binning, Timothée Poisot

**Affiliations:** 1 Université de Sherbrooke, Département de Biologie, Sherbrooke, Canada; 2 University of British Columbia, Faculty of Forestry, Vancouver, Canada; 3 University of Arizona, School of Natural Resources & Environment, Tucson, Arizona, United States of America; 4 Carleton University, Institute for Environmental & Interdisciplinary Science, Ottawa, Canada; 5 Université de Montréal, Département de Sciences Biologiques, Montréal, Canada

## Abstract

Post-doctoral scientists are effective mentors for graduate students. This Perspective discusses how failures to properly credit them for this role has negative consequence for everyone and suggests possible solutions.

## Teaser

Academia often fails to recognize the important work that supports its functioning, such as mentoring and teaching performed by postdoctoral researchers. This is a particular problem for early-career researchers, but opportunities exist to improve the status quo.

One adjective commonly applied to postdoctoral scientists (postdocs) and their work is “invisible”: invisible scholars [1], invisible innovators, invisible mentors. These metaphors describe the reality of the labor performed by many postdocs; these tasks are often expected of them by the demands of the academic job market but seldom formally credited to them. Owing to their (often) dual role as employees and trainees, they are expected to fulfill the obligations of both while reaping the benefits of neither. For example, postdocs are not always eligible to apply for independent research funding and, therefore, need to split credit with researchers with more job security. Moreover, when postdocs contribute to teaching, they are not always listed as instructors of record (designated as in charge of the course), and, therefore, this work may not be consistently recognized in job applications. They are also no longer eligible for training, grants, or fellowships exclusive for students, and in many institutions, postdocs do not have health benefits coverage, unlike graduate students and faculty, for whom coverage is often mandatory. For example, in the province of Québec, Canada, health insurance coverage for postdocs can vary according to immigration status, history of foreign residency, medical history, immigration status of spouse or common-law partner, intercountry agreements, specific job title, and university partnerships with private insurance companies. For postdocs wanting to pursue an academic career, this lack of recognition can put them at a serious disadvantage, as uncredited work can come at the expense of contributing to research projects.

This conundrum is particularly true when it comes to mentoring graduate students. Postdocs are highly trained, up-to-date on the literature, have a fresh eye on the state of the art in their field, and are often leading experts in emerging methodologies and approaches. In fact, data suggest that over a 5-year period, postdocs in the life sciences outpublish graduate students and faculty [2], which demonstrates their familiarity with cutting-edge scientific research subjects and practices. Being early in their career, they have likely very recently experienced the rigors of postgraduate education, putting them in a unique situation to establish rapport with mentees and provide practical advice for navigating the constantly evolving norms and practices of the academic world. Therefore, graduate students can benefit tremendously from being formally paired with postdocs as part of their supervisory team. In fact, research indicates that, in labs where postdocs are more engaged, PhD students can be 4 times as likely to have positive skill development; mentoring by principal investigators had no discernible statistical effect on the same variable [3].

Yet, in practice, the contribution of postdocs in training graduate students frequently remains invisible; the mentoring happens but often without explicit credit to the postdocs. As with all invisible tasks, they add work hours on top of the time spent on research, which can lead to burnout and frustration. This issue, along with most other labor-related questions in academia, intersects with additional burdens and biases about gender and race [4–6]. A lack of financial compensation, along with a lack of institutional and supervisor acknowledgement that postdocs often have non-work-related responsibilities (e.g., dependents in their care), places additional undue strain on scientists during this vulnerable career stage [7].

An obvious way to challenge this status quo is to formally recognize and, when appropriate, encourage effective mentoring of graduate students by postdocs, by implementing policies to support (or striking down policies that prevent), and formally acknowledge, these mentoring relationships (Box 1). This approach would bring considerable benefits to the academic workforce and student body. Postdocs would gain experience in advising students, which prepares them to be more effective advisors, managers, and leaders in the future; this skill is invaluable both in academic and nonacademic careers, where former postdocs will likely have some managerial or leadership responsibilities. Graduate students would benefit through the diversification of their mentors and, in particular, could choose to be cosupervised by academics from a more diverse pool than faculty [8]; diversity in mentorship can be particularly important for graduate students from underrepresented minorities, who can often find a role model in their postdoc coadvisor. Faculty advisors would get to share some of their advising experience with postdocs while also benefiting from the complementary viewpoint of another advisor with a different lived experience of academia. Notably, by formally sharing their supervision and mentoring duties with postdocs, faculty can work towards avoiding burnout and overworking, preventing academic frustration and quitting [9].

**Box 1. Possible solutions to make visible the “invisible work” performed by postdocs**

**Policy**

- Government funding agencies should include postdocs as an eligible category of coauthorship in grant applications. This would benefit the postdoc, as they would gain skills in grant writing, project management, and leadership. It is also beneficial for research groups, as the chances of retaining the postdoc rise, and for funding agencies, for whom the diversity of their grantees increases. Grant regulations should clearly outline the role of various researchers in a way that accounts for the possibility of postdocs changing institutions (this would clarify the rules for all researchers, as faculty also change institutions).
- Another important step is to professionalize the employment of postdocs by ensuring their position is that of an employee rather than a student (in Brazil, for example, postdocs are considered to be students by funding agencies and are exempt from workers’ protections and ineligible for social security benefits). By professionalizing the postdoc position, a scientist’s relationship with their institution becomes a protected mutual agreement that incurs more professional security.

**Institutions**

- Institutions often have the power to design postdoc positions, therefore determining postdocs’ rights and obligations. It is crucial that postdoc appointments clearly designate their relationship with the institution, notably by removing obligations that are typically expected from students (such as paying tuition).
- Institutions should require research groups to explicitly define postdocs’ rights and obligations on job offers and should allow postdocs to be listed as a member of a students’ supervision committee so that they can receive mentoring credit.
- It should be mandatory that all postdocs are listed as an instructor of record in the courses they are the lead instructor for.
- Institutions should include mentoring experience in promotion and tenure assessments to incentivize reward participation and personal investment in these activities.

**Research groups**

- At the level of research groups that are hiring postdocs, it is essential that principal investigators are very clear about expectations. If they expect postdocs to mentor, teach, or manage students, these activities should be listed in the job advertisement, including the expected amount of required time for each. After making an offer, group leaders should discuss with the candidate if they are interested in any of these activities and adjust the offer letter to reflect their agreement. Once hired, postdocs should feel safe to stick to the agreement and not feel obligated to work overtime.
- It is the obligation of the research group leader to guarantee a safe environment for their postdocs.

The purpose of postdoctoral training is to acquire core skills that will be useful in an academic’s next career stage. Putting into practice policies that formally recognize scientific advising (or any other labor not properly credited, such as management, applying for grants, and teaching) might require that faculty revise their expectations of a postdoctoral position. Additionally, internal policies should be explicit about what postdocs can do, which would help them make informed decisions early in their application for the job. Conversely, postdocs should not be expected to perform supervision and mentoring if these tasks are not clearly delineated in their contracts, if this is not a skill they wish to acquire, or if they stand no chance of receiving credit and recognition for them. For this to be achieved, faculty and staff might need to negotiate with unions and associations to protect postdocs’ rights to perform some of these tasks [10].

As mentoring often involves more than one institution, there also needs to be a cultural change on a cross-institutional level. Postdocs should be able to formalize their mentoring activities, both in their home institution and their advisee’s, and they should also be recognized as independent researchers by funding agencies and scientific journals. Although all the tasks performed by postdocs can be freely stated on their CV, formal recognition would give them power to act independently and take responsibility in bureaucratic rituals, such as sitting in committee meetings and qualifying exams, signing grant proposals as coapplicants, and being listed in joint last authorship on papers from cosupervised students. In certain situations, this formal recognition may be necessary to apply for academic teaching positions and accreditation.

It takes a village to mentor a graduate student; it makes sense that this village is not solely inhabited by faculty, or that faculty alone should be able to claim the credit for the mentoring process. Proper attribution of intellectual credit is a cornerstone of academic reputation building [11]; in this context, the time, energy, and skill invested by postdocs in the mentoring and advising of graduate mentees must be recognized, as not doing so robs them of credit for the important work they perform. Neglecting to recognize and credit mentoring and advising as part of a postdoc’s job is a disservice to science, research, and education.

## References

[1] Jaeger A, Dinin AJ, editors. The Postdoc Landscape. Academic Press; 2018. doi: 10.1016/C2016-0-04017-2

[2] Cook I, Grange S, Eyre-Walker A. Research groups: How big should they be? PeerJ. 2015;3:e989. doi: 10.7717/peerj.989 26082872 PMC4465944

[3] Feldon DF, Litson K, Jeong S, Blaney JM, Kang J, Miller C, et al. Postdocs’ lab engagement predicts trajectories of PhD students’ skill development. Proc Natl Acad Sci U S A. 2019;116:20910–20916. doi: 10.1073/pnas.1912488116 31570599 PMC6800364

[4] Case SS, Richley BA. Gendered institutional research cultures in science: the post-doc transition for women scientists. Community Work Fam. 2013;16:327–349. doi: 10.1080/13668803.2013.820097

[5] Eaton AA, Saunders JF, Jacobson RK, West K. How Gender and Race Stereotypes Impact the Advancement of Scholars in STEM: Professors’ Biased Evaluations of Physics and Biology Post-Doctoral Candidates. Sex Roles. 2019;82:127–141. doi: 10.1007/s11199-019-01052-w

[6] Moss-Racusin CA, Dovidio JF, Brescoll VL, Graham MJ, Handelsman J. Science faculty’s subtle gender biases favor male students. Proc Natl Acad Sci U S A. 2012;109:16474–16479. doi: 10.1073/pnas.1211286109 22988126 PMC3478626

[7] Forrester N. Fed up and burnt out: ‘quiet quitting’ hits academia. Nature. 2023;615:751–753. doi: 10.1038/d41586-023-00633-w 36869111

[8] Deanna R, Merkle BG, Chun KP, Navarro-Rosenblatt D, Baxter I, Oleas N, et al. Community voices: the importance of diverse networks in academic mentoring. Nat Commun. 2022;13:1681. doi: 10.1038/s41467-022-28667-0 35338138 PMC8956734

[9] Rahal R-M, Fiedler S, Adetula A, Berntsson RP-A, Dirnagl U, Feld GB, et al. Quality research needs good working conditions. Nat Hum Behav. 2023;7:164–167. doi: 10.1038/s41562-022-01508-2 36755134

[10] Ålund M, Emery N, Jarrett BJM, MacLeod KJ, McCreery HF, Mamoozadeh N, et al. Academic ecosystems must evolve to support a sustainable postdoc workforce. Nat Ecol Evol. 2020;4:777–781. doi: 10.1038/s41559-020-1178-6 32284583

[11] Thurston MM, Moniri NH, Bowen JP, Winkles CL, Miller SW. Managing the Three Cs of Academic Literature Authorship: Contributions Credit, and Conflict. Am J Pharm Educ. 2023;87:100009. doi: 10.1016/j.ajpe.2022.10.002 37288678

